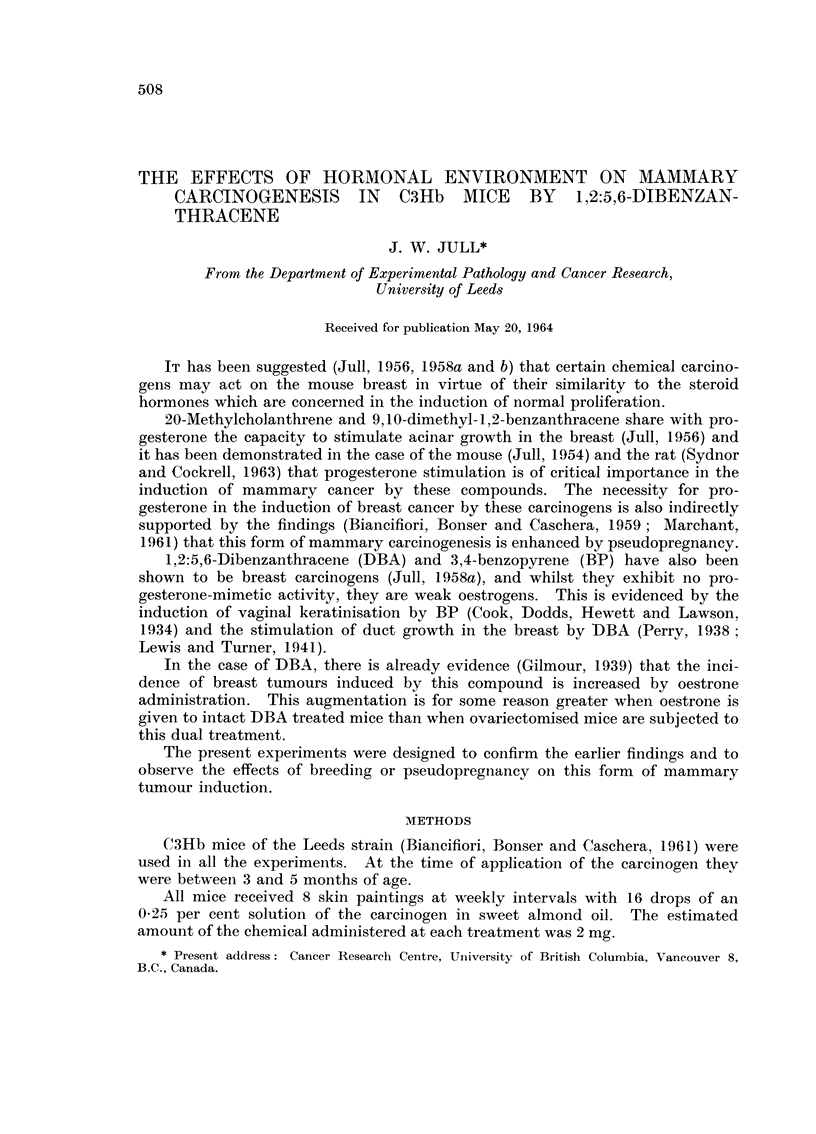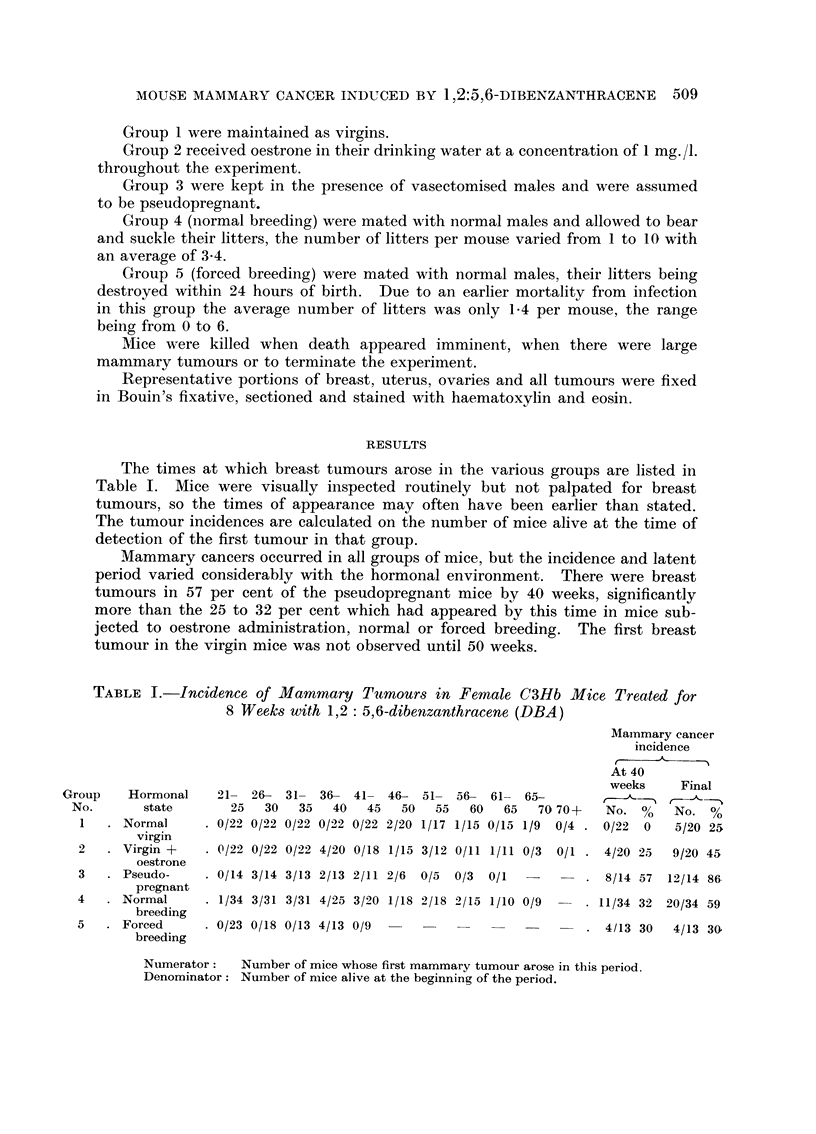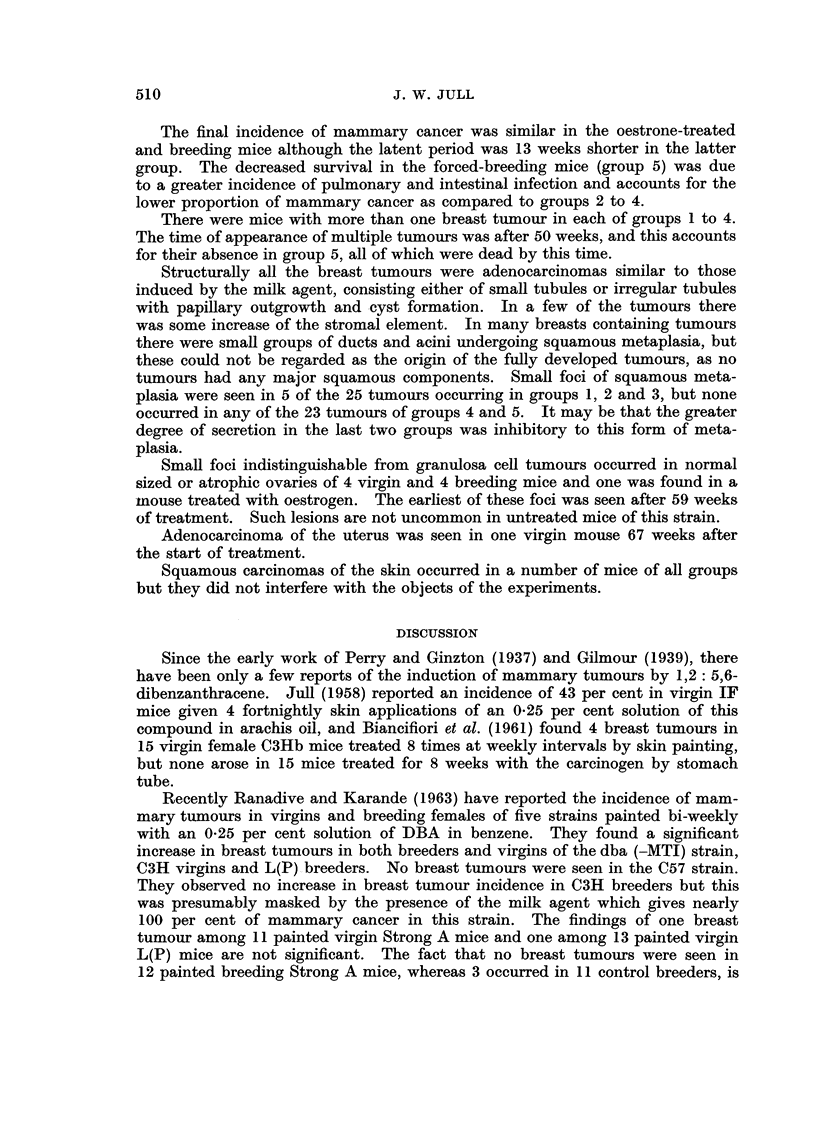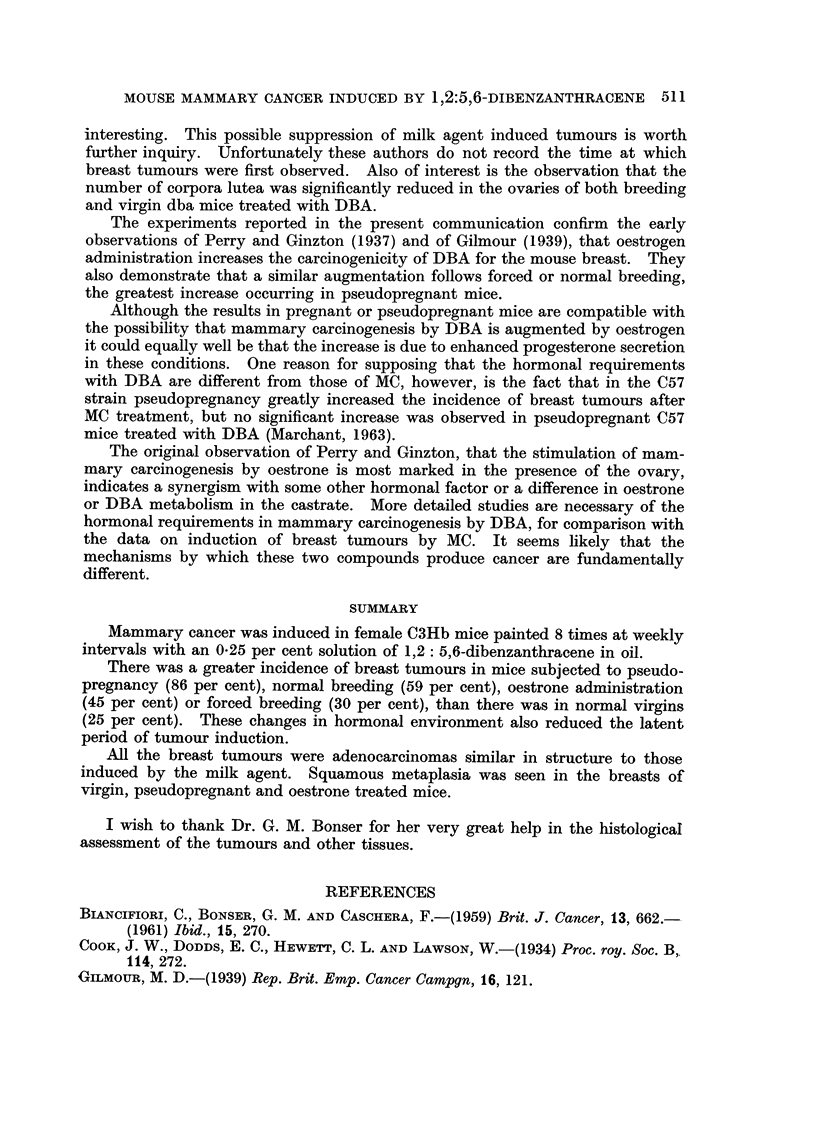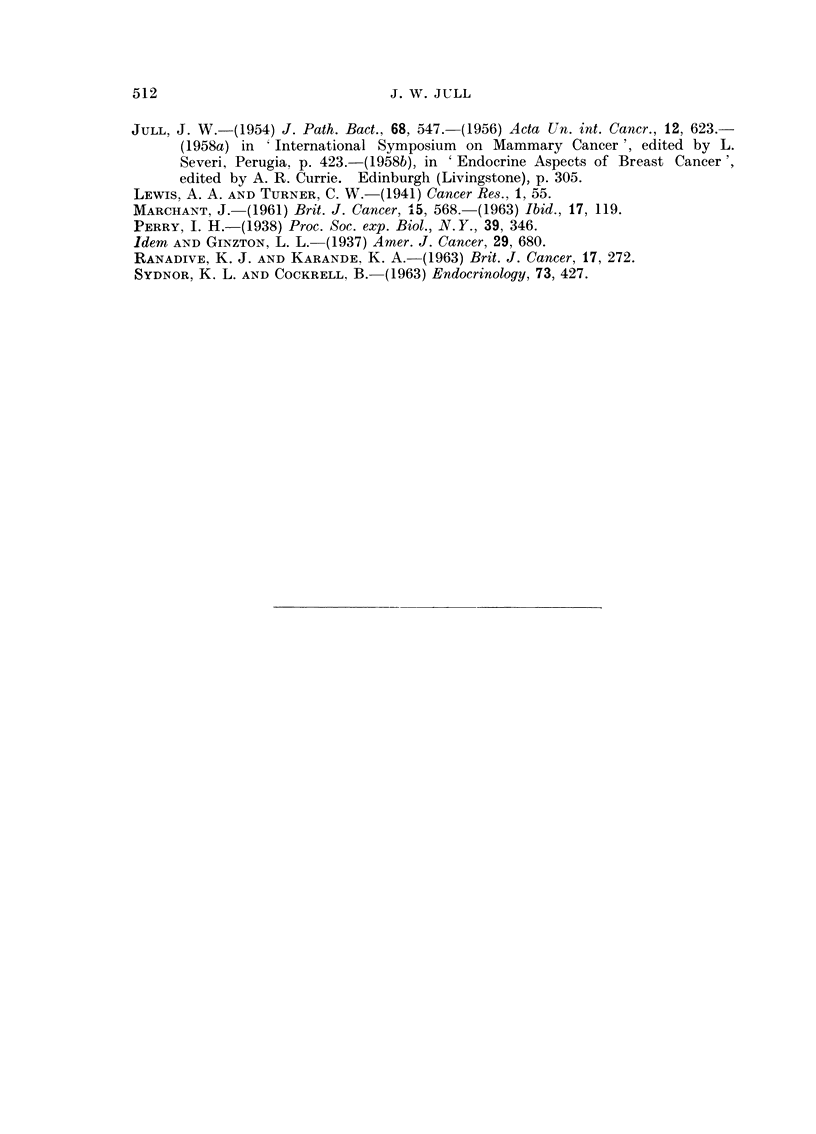# The Effects of Hormonal Environment on Mammary Carcinogenesis in C3Hb Mice by 1,2:5,6-Dibenzanthracene

**DOI:** 10.1038/bjc.1964.57

**Published:** 1964-09

**Authors:** J. W. Jull


### THE EFFECTS OF HORMONAL ENVIRONMENT ON MAMMARY CARCINOGENESIS IN C3Hb MICE BY 1,2:5,6-DIBENZANTHRACENE

J. W. JULL*

*From the Department of Experimental Pathology and Cancer Research, University of Leeds*

Received for publication May 20, 1964

IT has been suggested (Jull, 1956, 1958*a* and *b*) that certain chemical carcinogens may act on the mouse breast in virtue of their similarity to the steroid hormones which are concerned in the induction of normal proliferation.

20-Methylcholanthrene and 9,10-dimethyl-1,2-benzanthracene share with progesterone the capacity to stimulate acinar growth in the breast (Jull, 1956) and it has been demonstrated in the case of the mouse (Jull, 1954) and the rat (Sydnor and Cockrell, 1963) that progesterone stimulation is of critical importance in the induction of mammary cancer by these compounds. The necessity for progesterone in the induction of breast cancer by these carcinogens is also indirectly supported by the findings (Biancifiori, Bonser and Caschera, 1959; Marchant, 1961) that this form of mammary carcinogenesis is enhanced by pseudopregnancy.

1,2:5,6-Dibenzanthracene (DBA) and 3,4-benzopyrene (BP) have also been shown to be breast carcinogens (Jull, 1958*a*), and whilst they exhibit no progesterone-mimetic activity, they are weak oestrogens. This is evidenced by the induction of vaginal keratinisation by BP (Cook, Dodds, Hewett and Lawson, 1934) and the stimulation of duct growth in the breast by DBA (Perry, 1938; Lewis and Turner, 1941).

In the case of DBA, there is already evidence (Gilmour, 1939) that the incidence of breast tumours induced by this compound is increased by oestrone administration. This augmentation is for some reason greater when oestrone is given to intact DBA treated mice than when ovariectomised mice are subjected to this dual treatment.

The present experiments were designed to confirm the earlier findings and to observe the effects of breeding or pseudopregnancy on this form of mammary tumour induction.

### METHODS

C3Hb mice of the Leeds strain (Biancifiori, Bonser and Caschera, 1961) were used in all the experiments. At the time of application of the carcinogen they were between 3 and 5 months of age.

All mice received 8 skin paintings at weekly intervals with 16 drops of an 0·25 per cent solution of the carcinogen in sweet almond oil. The estimated amount of the chemical administered at each treatment was 2 mg.

* Present address: Cancer Research Centre, University of British Columbia, Vancouver 8, B.C., Canada.

Group 1 were maintained as virgins.

Group 2 received oestrone in their drinking water at a concentration of 1 mg./l. throughout the experiment.

Group 3 were kept in the presence of vasectomised males and were assumed to be pseudopregnant.

Group 4 (normal breeding) were mated with normal males and allowed to bear and suckle their litters, the number of litters per mouse varied from 1 to 10 with an average of 3·4.

Group 5 (forced breeding) were mated with normal males, their litters being destroyed within 24 hours of birth. Due to an earlier mortality from infection in this group the average number of litters was only 1·4 per mouse, the range being from 0 to 6.

Mice were killed when death appeared imminent, when there were large mammary tumours or to terminate the experiment.

Representative portions of breast, uterus, ovaries and all tumours were fixed in Bouin's fixative, sectioned and stained with haematoxylin and eosin.

### RESULTS

The times at which breast tumours arose in the various groups are listed in Table I. Mice were visually inspected routinely but not palpated for breast tumours, so the times of appearance may often have been earlier than stated. The tumour incidences are calculated on the number of mice alive at the time of detection of the first tumour in that group.

Mammary cancers occurred in all groups of mice, but the incidence and latent period varied considerably with the hormonal environment. There were breast tumours in 57 per cent of the pseudopregnant mice by 40 weeks, significantly more than the 25 to 32 per cent which had appeared by this time in mice subjected to oestrone administration, normal or forced breeding. The first breast tumour in the virgin mice was not observed until 50 weeks.

TABLE I.—*Incidence of Mammary Tumours in Female C3Hb Mice Treated for 8 Weeks with 1,2 : 5,6-dibenzanthracene (DBA)*

| | | | | | | | | | | | | | Mammary cancer incidence | | | |
|---|---|---|---|---|---|---|---|---|---|---|---|---|---|---|---|---|
| | | | | | | | | | | | | | At 40 weeks | | Final | |
| Group No. | Hormonal state | 21–25 | 26–30 | 31–35 | 36–40 | 41–45 | 46–50 | 51–55 | 56–60 | 61–65 | 65–70 | 70+ | No. | % | No. | % |
| 1 | Normal virgin | 0/22 | 0/22 | 0/22 | 0/22 | 0/22 | 2/20 | 1/17 | 1/15 | 0/15 | 1/9 | 0/4 | 0/22 | 0 | 5/20 | 25 |
| 2 | Virgin + oestrone | 0/22 | 0/22 | 0/22 | 4/20 | 0/18 | 1/15 | 3/12 | 0/11 | 1/11 | 0/3 | 0/1 | 4/20 | 25 | 9/20 | 45 |
| 3 | Pseudo-pregnant | 0/14 | 3/14 | 3/13 | 2/13 | 2/11 | 2/6 | 0/5 | 0/3 | 0/1 | — | — | 8/14 | 57 | 12/14 | 86 |
| 4 | Normal breeding | 1/34 | 3/31 | 3/31 | 4/25 | 3/20 | 1/18 | 2/18 | 2/15 | 1/10 | 0/9 | — | 11/34 | 32 | 20/34 | 59 |
| 5 | Forced breeding | 0/23 | 0/18 | 0/13 | 4/13 | 0/9 | — | — | — | — | — | — | 4/13 | 30 | 4/13 | 30 |

Numerator : Number of mice whose first mammary tumour arose in this period.
Denominator : Number of mice alive at the beginning of the period.

The final incidence of mammary cancer was similar in the oestrone-treated and breeding mice although the latent period was 13 weeks shorter in the latter group. The decreased survival in the forced-breeding mice (group 5) was due to a greater incidence of pulmonary and intestinal infection and accounts for the lower proportion of mammary cancer as compared to groups 2 to 4.

There were mice with more than one breast tumour in each of groups 1 to 4. The time of appearance of multiple tumours was after 50 weeks, and this accounts for their absence in group 5, all of which were dead by this time.

Structurally all the breast tumours were adenocarcinomas similar to those induced by the milk agent, consisting either of small tubules or irregular tubules with papillary outgrowth and cyst formation. In a few of the tumours there was some increase of the stromal element. In many breasts containing tumours there were small groups of ducts and acini undergoing squamous metaplasia, but these could not be regarded as the origin of the fully developed tumours, as no tumours had any major squamous components. Small foci of squamous metaplasia were seen in 5 of the 25 tumours occurring in groups 1, 2 and 3, but none occurred in any of the 23 tumours of groups 4 and 5. It may be that the greater degree of secretion in the last two groups was inhibitory to this form of metaplasia.

Small foci indistinguishable from granulosa cell tumours occurred in normal sized or atrophic ovaries of 4 virgin and 4 breeding mice and one was found in a mouse treated with oestrogen. The earliest of these foci was seen after 59 weeks of treatment. Such lesions are not uncommon in untreated mice of this strain.

Adenocarcinoma of the uterus was seen in one virgin mouse 67 weeks after the start of treatment.

Squamous carcinomas of the skin occurred in a number of mice of all groups but they did not interfere with the objects of the experiments.

### DISCUSSION

Since the early work of Perry and Ginzton (1937) and Gilmour (1939), there have been only a few reports of the induction of mammary tumours by 1,2 : 5,6-dibenzanthracene. Jull (1958) reported an incidence of 43 per cent in virgin IF mice given 4 fortnightly skin applications of an 0·25 per cent solution of this compound in arachis oil, and Biancifiori *et al.* (1961) found 4 breast tumours in 15 virgin female C3Hb mice treated 8 times at weekly intervals by skin painting, but none arose in 15 mice treated for 8 weeks with the carcinogen by stomach tube.

Recently Ranadive and Karande (1963) have reported the incidence of mammary tumours in virgins and breeding females of five strains painted bi-weekly with an 0·25 per cent solution of DBA in benzene. They found a significant increase in breast tumours in both breeders and virgins of the dba (–MTI) strain, C3H virgins and L(P) breeders. No breast tumours were seen in the C57 strain. They observed no increase in breast tumour incidence in C3H breeders but this was presumably masked by the presence of the milk agent which gives nearly 100 per cent of mammary cancer in this strain. The findings of one breast tumour among 11 painted virgin Strong A mice and one among 13 painted virgin L(P) mice are not significant. The fact that no breast tumours were seen in 12 painted breeding Strong A mice, whereas 3 occurred in 11 control breeders, is interesting. This possible suppression of milk agent induced tumours is worth further inquiry. Unfortunately these authors do not record the time at which breast tumours were first observed. Also of interest is the observation that the number of corpora lutea was significantly reduced in the ovaries of both breeding and virgin dba mice treated with DBA.

The experiments reported in the present communication confirm the early observations of Perry and Ginzton (1937) and of Gilmour (1939), that oestrogen administration increases the carcinogenicity of DBA for the mouse breast. They also demonstrate that a similar augmentation follows forced or normal breeding, the greatest increase occurring in pseudopregnant mice.

Although the results in pregnant or pseudopregnant mice are compatible with the possibility that mammary carcinogenesis by DBA is augmented by oestrogen it could equally well be that the increase is due to enhanced progesterone secretion in these conditions. One reason for supposing that the hormonal requirements with DBA are different from those of MC, however, is the fact that in the C57 strain pseudopregnancy greatly increased the incidence of breast tumours after MC treatment, but no significant increase was observed in pseudopregnant C57 mice treated with DBA (Marchant, 1963).

The original observation of Perry and Ginzton, that the stimulation of mammary carcinogenesis by oestrone is most marked in the presence of the ovary, indicates a synergism with some other hormonal factor or a difference in oestrone or DBA metabolism in the castrate. More detailed studies are necessary of the hormonal requirements in mammary carcinogenesis by DBA, for comparison with the data on induction of breast tumours by MC. It seems likely that the mechanisms by which these two compounds produce cancer are fundamentally different.

### SUMMARY

Mammary cancer was induced in female C3Hb mice painted 8 times at weekly intervals with an 0·25 per cent solution of 1,2 : 5,6-dibenzanthracene in oil.

There was a greater incidence of breast tumours in mice subjected to pseudopregnancy (86 per cent), normal breeding (59 per cent), oestrone administration (45 per cent) or forced breeding (30 per cent), than there was in normal virgins (25 per cent). These changes in hormonal environment also reduced the latent period of tumour induction.

All the breast tumours were adenocarcinomas similar in structure to those induced by the milk agent. Squamous metaplasia was seen in the breasts of virgin, pseudopregnant and oestrone treated mice.

I wish to thank Dr. G. M. Bonser for her very great help in the histological assessment of the tumours and other tissues.

### REFERENCES

BIANCIFIORI, C., BONSER, G. M. AND CASCHERA, F.—(1959) *Brit. J. Cancer*, **13**, 662.—(1961) *Ibid.*, **15**, 270.

COOK, J. W., DODDS, E. C., HEWETT, C. L. AND LAWSON, W.—(1934) *Proc. roy. Soc.* B, **114**, 272.

GILMOUR, M. D.—(1939) *Rep. Brit. Emp. Cancer Campgn*, **16**, 121.

JULL, J. W.—(1954) *J. Path. Bact.*, **68**, 547.—(1956) *Acta Un. int. Cancr.*, **12**, 623.—(1958*a*) in ' International Symposium on Mammary Cancer ', edited by L. Severi, Perugia, p. 423.—(1958*b*), in ' Endocrine Aspects of Breast Cancer ', edited by A. R. Currie. Edinburgh (Livingstone), p. 305.
LEWIS, A. A. AND TURNER, C. W.—(1941) *Cancer Res.*, **1**, 55.
MARCHANT, J.—(1961) *Brit. J. Cancer*, **15**, 568.—(1963) *Ibid.*, **17**, 119.
PERRY, I. H.—(1938) *Proc. Soc. exp. Biol., N.Y.*, **39**, 346.
*Idem* AND GINZTON, L. L.—(1937) *Amer. J. Cancer*, **29**, 680.
RANADIVE, K. J. AND KARANDE, K. A.—(1963) *Brit. J. Cancer*, **17**, 272.
SYDNOR, K. L. AND COCKRELL, B.—(1963) *Endocrinology*, **73**, 427.